# Supplementary material for: The Impact of Falls: A Qualitative Study of the Experiences of People Receiving Haemodialysis
Source: Int J Environ Res Public Health. 2022 Mar 24;19(7):3873. doi: 10.3390/ijerph19073873 (PMC8997574; doi:10.3390/ijerph19073873)
Supplement: Supplementary file 1 [file ijerph-19-03873-s001.zip › Supplementary falls material 1.pdf]

Supplementary material 1: Final topic guide used within the interview

1. To begin, can you tell me a little bit about how you came to be on dialysis?

2. Has dialysis affected you physically?

Prompts:

- -Weakness,
- -Slowness,
- -Exhaustion,
- -Levels of physical activity
- -Unexplained weight loss

3. Has dialysis affected you in any other ways?

Prompts – changes/challenges to:

- Daily routine
- Health, other long term conditions
- Interactions with healthcare professionals
- Needing help with ADLS/finance/medication
- Social interactions and hobbies
- Cognition, concentration and memory
- Psychological – happiness, loneliness, emotion, outlook on life, confidence

4. One of the reasons I asked you to take part in this study is because you have had a fall in the past. Can you tell me about what happened when you last fell?

Prompts:

- Do they remember?
- dizziness/ unsteadiness/ trips and slips/ vertigo/ syncope
- Activity at time?
- Direction of fall?
- Where? Probe into environment factors involved
- Time of day?
- Alone or accompanied?
- Injury?
- Able to get up?
- Pendant alarm

5. What do you think caused you to fall over?

- Environment (home, dialysis, outside)
- Post dialysis effects
- Cardiac impairment
- Balance impairment
- Neurological cause
- ENT impairment

- External force
- Lack of activity/ weakness
- Pre-existing health problem
- Medication

Did others have different ideas of cause?

6. What happened immediately after you fell?
   - What self-care did they undertake?
   - Healthcare utilisation/ contact with health professional or other service
   - Family response/ impact
   - Social impact / impact on daily life and routine
   - Emotional impact / impact on confidence
   - Physical impact – health/ activity levels/ function

7. What happened because of the fall in the longer term?

- Same prompts as question 6

8. Did you discuss this fall anyone at the time?

Prompts:

If yes – who [Healthcare professionals, family friends etc.]?

- Who initiated this and why
- What was discussed, with whom, outcome?

If no – why not?

9. Were you offered any support or treatment after you fell?

Yes – what support was offered?

- Thoughts on what was offered/ advised/ suggested?
- Did they take it?
- Was the outcome?
- Did the face any challenges to participating/accessing this treatment or support?

No/ did not take – why not?

- What do you think would have been useful?
