# Supplementary material for: The Impact of Falls: A Qualitative Study of the Experiences of People Receiving Haemodialysis
Source: Int J Environ Res Public Health. 2022 Mar 24;19(7):3873. doi: 10.3390/ijerph19073873 (PMC8997574; doi:10.3390/ijerph19073873)
Supplement: Supplementary file 1 [file ijerph-19-03873-s001.zip › supplementary falls material 2.pdf]

Supplementary material 2. Outline of the diary-keeping information booklet provided to participants

Section of the diary information booklet	Information provided
**Aim**	Outline of why diaries are being sought.
**Introduction**	Legitimisation of forgetting to record and not recording daily.
**What should I record?**	• Falls: plain English definition of fall provided.<br>• Specific information of interest outlined in detail.<br>• Encouragement to record anything of interest, no matter how seemingly trivial.<br>• A reminder not to worry about spelling, handwriting or grammar if keeping a written diary.
**Example diary entries**	• Examples of diary entries included.
**What will happen whilst I am keeping my diary?**	• Support offered during diary-keeping phase outlined.
**What will happen when I have finished keeping my diary?**	• Details of how diary would be collected, and what to do if they wished to finish early.<br>• Information on interview and secure storage arrangements for diary data provided
**What should I do if I need some help?**	• Researcher contact details provided.<br>• Participants thanked for their involvement in the study.
